# Effect of the glucocorticoid receptor antagonist RU486 on MK-801 induced behavioural sensitisation

**DOI:** 10.1371/journal.pone.0176156

**Published:** 2017-04-21

**Authors:** Emilia M. Lefevre, Gregory A. Medley, Timothy Reeks, Suzy Alexander, Thomas H. J. Burne, Darryl W. Eyles

**Affiliations:** 1 Queensland Brain Institute, The University of Queensland, St Lucia, Queensland, Australia; 2 Queensland Centre for Mental Health Research, The Park Centre for Mental Health, Richlands, Queensland, Australia; University of South Carolina School of Medicine, UNITED STATES

## Abstract

Stress is known to modulate sensitisation to repeated psychostimulant exposure. However, there is no direct evidence linking glucocorticoids and sensitisation achieved by repeated administration of the NMDA receptor antagonist MK-801. We tested the hypothesis that co-administration of RU486, a glucocorticoid receptor (GR) antagonist, prior to repeated daily MK-801 injections would block the expression of locomotor sensitisation due to its dual effects on corticosterone and dopamine. We employed a repeated MK-801 administration locomotor sensitisation paradigm in male Sprague Dawley rats. RU486 or a dimethyl sulfoxide (DMSO) vehicle was co-administered with MK-801 or saline during the induction phase. Subsequent to withdrawal, rats were challenged with MK-801 alone to test for the expression of sensitisation. In a separate cohort of rats, plasma corticosterone levels were quantified from blood samples taken on the 1^st^, 4^th^ and 7^th^ day of induction and at expression. One day after challenge, nucleus accumbens tissue levels of dopamine and its metabolites DOPAC and HVA were measured. During the induction phase, RU486 progressively enhanced locomotor sensitisation to MK-801. RU486 and MK-801 both showed stimulatory effects on corticosterone levels and this was further augmented when given in combination. Contrary to our hypothesis, RU486 did not block the expression of locomotor sensitisation to MK-801 and actually increased levels of dopamine, DOPAC and HVA in nucleus accumbens tissue. Our results showed that RU486 has augmentative rather than inhibitory effects on MK-801-induced sensitisation. This study indicates a divergent role for glucocorticoids in sensitisation to MK-801 compared to sensitisation with other psychostimulants.

## Introduction

Sensitisation can be defined as the enhanced behavioural or neurochemical response to a drug following repeated psychostimulant exposure. In rodents, behavioural sensitisation is typically observed as an augmented hyperlocomotor response to a drug challenge after withdrawal from repeated drug administration. This phenomenon is typically studied to understand putative neurochemical adaptations that occur within drug abuse. Furthermore, the progressive and relapsing nature of sensitisation is thought to be a potential model for disorders such as drug addiction [1] and schizophrenia [2]. Behavioural sensitisation can be divided into two key phases, induction and expression, which are separated by a period of drug abstinence or withdrawal. The induction of sensitisation refers to the neurological changes that develop with repeated drug exposure, whereas the expression of sensitisation is characterized as the enhanced response to a drug challenge [3]. The ability of different classes of drugs of abuse to induce sensitisation is indicative of the multiple mechanisms involved in these behavioural and neurochemical adaptations [4].

Several lines of evidence indicate that N-methyl-D-aspartate (NMDA) receptor signalling plays a critical role in the development of behavioural sensitisation [5]. For example, the NMDA receptor antagonist MK-801 was found to block sensitisation to drugs of abuse including cocaine, amphetamine, morphine and ethanol [5]. Despite this, MK-801 is also known to produce behavioural and neurochemical sensitisation to its own effects following acute or repeated exposure [6]. This suggests that the mechanisms underlying sensitisation to MK-801 differ from other drugs of abuse, particularly dopamine agonists such as amphetamine and cocaine. In comparison to these dopaminergic psychostimulants, far less is known of the mechanisms mediating sensitisation to MK-801. The expression of behavioural sensitisation to most drugs of abuse is associated with enhanced mesoaccumbens dopamine activity [4]. Although the expression of MK-801 induced sensitisation is also associated with enhanced mesoaccumbens dopaminergic activity, locomotor sensitisation to MK-801 is not attenuated by dopamine receptor antagonism [6] as observed for other psychostimulants [4]. An important factor to take into consideration for the development of behavioural sensitisation is the environmental conditions and the stressful experiences associated with drug exposure. Contrary to data reported for other psychostimulants [7], we have shown that MK-801 induced behavioural sensitisation is not modulated by environmental context [8].

The hypothalamic-pituitary-adrenal (HPA) axis is activated in response to stress. Acute administration of drugs of abuse including cocaine [9], amphetamine [10], ethanol [11], morphine [12] and MK-801 [13] can also lead to HPA axis activation. Upon activation of the HPA axis corticosterone is released from the adrenal glands and signals at the high-affinity mineralocorticoid receptors (MR) and low-affinity glucocorticoid receptors (GR) [14]. Low basal corticosterone levels are believed to preferentially bind MR, whereas under stressful conditions increased corticosterone levels progressively saturate the GR [14, 15]. Research suggests that stress, via increased corticosterone signalling at the GRs, facilitates behavioural sensitisation to psychostimulant drugs.

The role of the glucocorticoids is thought to be predominately involved in the induction rather than expression phase of behavioural sensitisation. For instance it has been shown that adrenalectomy or the co-administration of the GR antagonist RU486 can block the induction of behavioural sensitisation to drugs of abuse [16–19]. However, once sensitisation is established, adrenalectomy past the induction phase has no effect on the expression of behavioural sensitisation [17, 19]. In agreement with these findings, co-administration of RU486 with drug challenge does not attenuate the expression of behavioural sensitisation [20]. Glucocorticoids are postulated to exert their effects on behavioural sensitisation through modulation of mesoaccumbens activity. For example, increased dopamine release in the NAc has been observed in the cross-sensitisation between stress and psychostimulant drugs in a glucocorticoid dependent manner [21–23]. Furthermore, the removal of corticosterone by adrenalectomy, or ablation of the GR has been shown to reduce psychostimulant-induced increases of dopamine release in the NAc [24, 25].

Corticosterone signalling via the GRs is thought to play a critical role in the induction of sensitisation to MK-801 [26]. Co-administration of the corticosterone synthesis inhibitor, metyrapone, was found to block induction of a sensitised locomotor response to a subsequent MK-801 challenge after two days withdrawal [26]. The authors also demonstrated that in their acute MK-801 sensitisation paradigm, co-administration of RU486 with the first injection MK-801 blocked the development of sensitisation [26]. Furthermore, acute administration of exogenous corticosterone can increase the hyperlocomotor stimulatory effects of MK-801 [26]. The manipulation of GRs during the induction of *repeated* MK-801 exposure has not previously been examined. Since acute MK-801 has activating effects on the HPA axis, this may, in part, mediate the ability of repeated MK-801 to induce behavioural sensitisation. Therefore, our primary objective in these experiments was to determine whether sensitisation to repeated MK-801 injections could be inhibited by co-administration of the GR antagonist RU486. In addition, how the induction and expression of MK-801 sensitisation interacted with GR antagonism to affect plasma corticosterone levels was examined. The mechanism by which glucocorticoids modulate NAc dopamine in a model of MK-801-induced sensitisation is currently unknown. Therefore, it was determined whether long-term changes in NAc dopamine were altered by MK-801 and/or RU486 administration. The two major metabolites of dopamine; 3,4-Dihydroxyphenylacetic acid (DOPAC) and homovanillic acid (HVA) were measured as useful indicators of dopamine release and turnover.

## Materials and methods

### Animals

Adult (~70 days of age) male Sprague Dawley rats (ARC, Western Australia) were utilised in this study. Rats were pair-housed in standard Makrolon wire-top cages (38x23.5x16cm), kept on a constant 12h light/dark cycle (light phase 0600h-1800h), and provided with standard rat chow (Specialty Feeds, Glen Forrest, Western Australia, Australia) and water *ad libitum*. Rats were acclimatized to these living conditions for a minimum period of one week prior to any behavioural testing. Both the homeroom and behavioural testing room were located in the same animal facility (Room Temp. 24°C, 40–60% Humidity). All testing was conducted during the light phase and at the same time of day across the sensitisation paradigm. Two separate cohorts of rats were used for the behavioural testing and plasma corticosterone analysis. All procedures were performed with the approval from the University of Queensland Animal Ethics Committee, and followed the Australian code for the care and use of animals for scientific purposes (NHMRC, 8^th^ Ed, 2013).

### Behavioural testing

Behavioural testing was conducted in a room separate to the homeroom of the animal facility. The behavioural room was also temperature controlled to the same conditions as the homeroom (room temperature 24°C, 40–60% humidity). Rats were transported in their home cages to the testing room at least half an hour prior to behavioural testing commenced to allow them to habituate to the new environment. Behavioural testing was conducted after at least 2 h into the light phase (0800h-1800h).

### Apparatus

The locomotor activity was assessed in twelve black Perspex chambers, 45cm x 45cm wide and 60cm deep. The chambers were illuminated by a central strip of LEDs (17 lux). The locomotor activity was recorded via a centrally placed video camera (IP8152 Vivotek, Taiwan). The total locomotor distance travelled was calculated using the Ethovision automated video tracking software (Noldus Information Technology, Wageningen, Netherlands) using differencing background subtraction to identify the rat. All test cages were cleaned with 70% ethanol between animals.

### Preparation of drugs and administration procedure

MK-801 (Sigma) was dissolved in 0.9% NaCl to a concentration of 0.25mg/ml. MK-801 was administered at a dose of 0.25mg/kg body weight. Animals were weighed daily and the injection volume was adjusted to body weight with 1ml/kg body weight. The GR antagonist RU486 (mifepristone; Cayman Chemicals, MI, USA) was dissolved in dimethyl sulfoxide (DMSO) to a concentration of 80mg/ml. This solution was administered at 0.25ml/kg body weight in order to give a dose of 20mg/kg body weight. DMSO (100%) was delivered as the vehicle control for RU486 and was also administered at 0.25ml/kg body weight. All solutions were administered via intraperitoneal (i.p.) injection using a 29-gauge needle (Terumo, Japan). Analytical reference solutions of corticosterone and the internal standard corticosterone-[9,11,12,12-2H4] were obtained from IsoSciences (King of Prussia, PA, USA). Stripped human plasma (VD-DC Mass Spec Gold) was obtained from Golden West Biologicals Inc. (Temecula, CA, USA). All solvents and reagents were of HPLC grade.

### Behavioural effects of RU486 in MK-801 sensitisation

**Fig 1:**
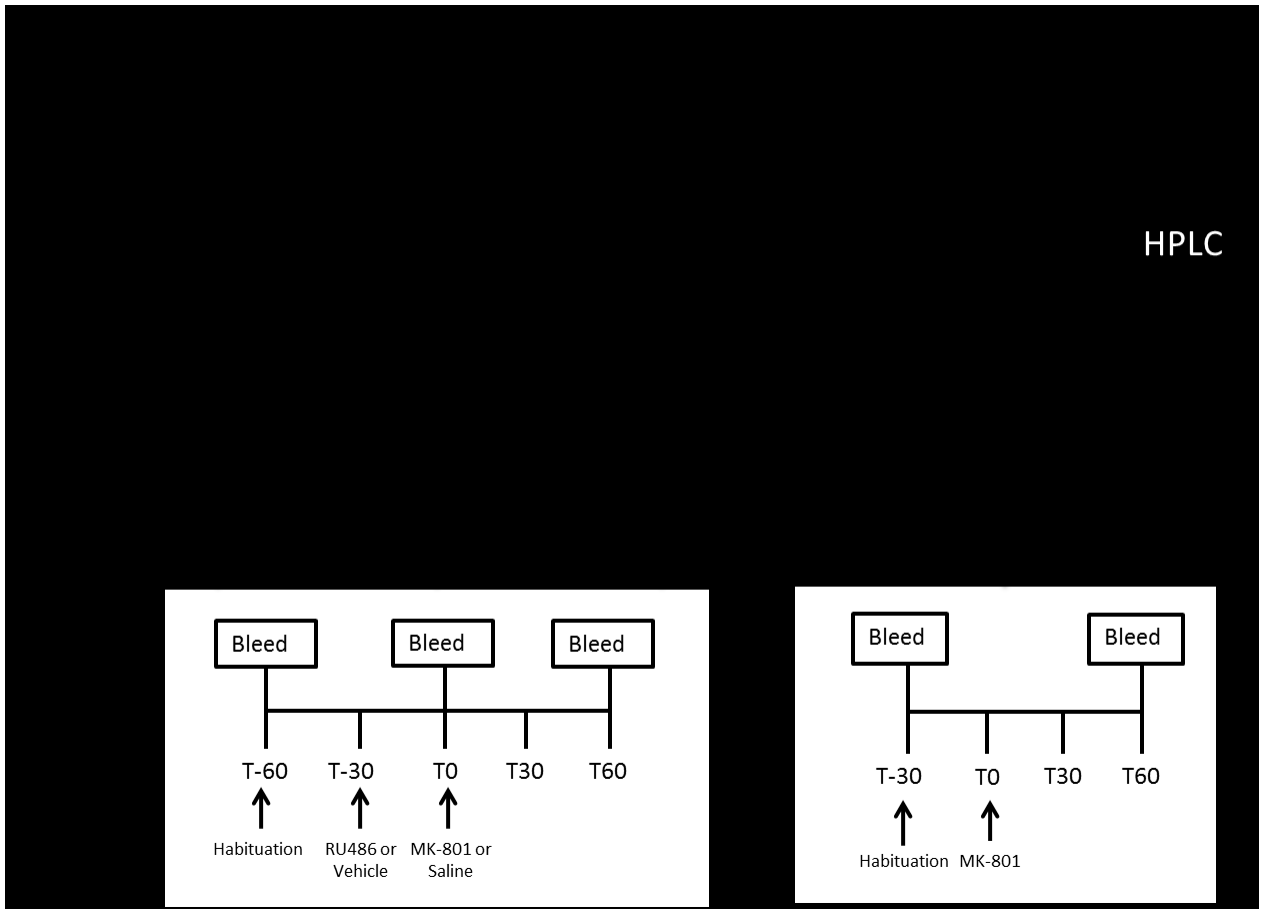
Experimental timeline. Timeline of the study. Behavioural testing was completed on the first cohort of animals (a) and corticosterone analysis on the second cohort (b). During the induction phase (Days 1–7) rats were sensitised with injections of MK-801 (0.25mg/kg) or Saline. Thirty min prior to these injections either RU486 (20mg/kg) or Vehicle (DMSO) was administered. In the second cohort blood samples were collected from the saphenous vein on Days 1, 4 and 7. The time points are relative to the MK-801 or saline injection at T0. The first sample was collected at T-60 min prior to habituation to the test chamber. After 30 min habituation (T-30) rats were injected with RU486 or Vehicle and the next sample was taken after 30min at T0. Rats were then injected with MK-801 or Saline and the third blood sample was collected 60min later at T60. At day 13, all rats were administered MK-801 to test for the expression of sensitisation. The first blood sample was taken at T-30, prior to habitation to the test chamber. At T0 rats were administered with MK-801 and the second blood sample was collected 60min later at T60.

The behavioural sensitisation paradigm used has previously been shown to induce locomotor sensitisation in Sprague Dawley rats (Lefevre et al., 2015) and is depicted in Fig 1a. Rats were injected once daily with MK-801 (0.25mg/kg) or saline (1ml/kg) over a 7-day induction phase. This was followed by a 5-day withdrawal phase in which rats were left untreated in their homecages. Following this, at the expression phase (Day 13) all rats received a challenge injection of MK-801 (0.25mg/kg). On each day of the induction phase, rats were habituated to the behavioural testing room for 30min. Once placed in the locomotor chamber, they were allowed another 30min habituation period. To investigate the role of GR in MK-801 sensitisation, the antagonist RU486 (20mg/kg) (n = 8/group) or a DMSO vehicle (n = 4/group) was administered on each day of the induction phase. RU486 or vehicle was administered after the 30min habituation phase and rats were immediately returned to the test chamber. After a further 30min, by which time RU486 concentrations were maximal [27], rats received the MK-801 or saline injection as per the sensitisation protocol. Locomotor activity was recorded for a further 120min. At the expression phase rats were habituated to the behavioural testing room for 30min followed by 30min habitation to the locomotor chamber. All rats were then challenged with an injection of MK-801 (0.25mg/kg) and locomotor activity recorded for 120min.

### Corticosterone effects of RU486 in MK-801 sensitisation

In a separate cohort of animals (n = 12/group), the effects of MK-801 and RU486 on plasma corticosterone signalling were examined in the same sensitisation protocol as described above. Corticosterone levels were assessed from blood samples taken on Day 1, 4, 7 of the induction phase and at the expression phase (Day 13). On Days 1, 4 and 7 blood was collected at three different time points, T-60, T0 and T60. As per the above protocol, rats were habituated to the behavioural testing room for 30min. Subsequent to this initial habituation period to the behavioural testing room, a baseline blood sample was collected (T-60). Rats were placed into the test cage and allowed a 30min habituation period. They were then administered an RU486 or DMSO vehicle injection and returned to the test cage. After 30min, the second blood sample was collected at the time point denoted as T0. Immediately after the blood sample was taken, rats received their MK-801 or saline injection. They were then returned to the test cage. At T60, the third blood sample was taken, hence 1h following MK-801 or saline administration and 1.5h after RU486 or vehicle administration. This protocol is described in Fig 1b.

### Blood sampling

Blood samples were collected from the saphenous vein by making a small prick with a needle (23 gauge, Nipro, NSW, Australia). The blood sample was taken while rats were gently restrained, the procedure took a maximum of 2min. Approximately 100ul of blood was collected into EDTA-coated tubes (Sarstedt, Nümbrecht, Germany). Samples were placed on ice and plasma was obtained by centrifugation at 1000RPM at 4°C for 10min. Plasma was stored at -20°C until assayed.

### Plasma corticosterone quantification

Corticosterone levels were quantified by an in-house Liquid Chromatography/ Tandem Mass Spectrometry (LC-MS/MS) technique. The system consisted of a Shimadzu Nexera^®^ UPLC system with a Phenomenex Kinetex^®^ 1.7u XB-C_18_ 100Å (50x2.1mm) column attached to an ABSciex QTrap-5500^®^ triple-quadrupole mass spectrometer. To 20μL aliquots of plasma, 20μL of internal standard (500 nM corticosterone-[^2^H_4_] in 1:1 acetonitrile: water), 10μL of 1M ZnSO_4_ and 600μL 9:1 ethyl-acetate: acetonitrile was added. After mixing, 500μL of the organic phase was decanted, dried under vacuum and reconstituted in 50μL of 1:1 methanol: water. 20μL of extract was then injected. A gradient elution method at 0.5mL/min was used with the mobile phases A = 0.1% aqueous formic acid and B = 0.1% formic acid in 9:1 acetonitrile: water. The mixture was increased from 50%B to 95%B over 2 min, held at 95%B for 0.5 min and then returned to 50%B for 1min. This resulted in a retention time of 1.2min. The mass-spectrometer detection was by way of positive-mode, scheduled multiple reaction monitoring with electrospray ionisation. The mass spectrometer parameters were as follows: for corticosterone: m/z = 247.1 → 329.1, declustering potential (DP) = 100V, exit potential (CXP) = 12, collision energy (CE) = 23V; for corticosterone-[^2^H_4_]: m/z = 351.1 → 333.0, DP = 100, CXP = 15, CE = 23. Calibration standards over the range 10—1000nM and quality controls at three levels were prepared in stripped plasma. Differential quality control samples were prepared by spiking rat serum with 75nM of corticosterone. A ±15% acceptance criterion for imprecision was applied to all quality controls.

### Dopamine and metabolites

Animals from the second cohort, from which plasma corticosterone was analysed, were euthanized with an overdose of Lethabarb (4ml/kg, i.p.) (Virbac Pty. Ltd., Australia) on Day 14. Tissue was collected 24h after the challenge injection of MK-801 to allow sufficient drug-washout [28]. The brains were dissected on an ice-chilled petri dish to collect the NAc region according to the method described by Carlsson and Lindqvist [29], then snap frozen in liquid nitrogen and stored at -80°C pending analysis. The NAc tissue was dispersed by sonication in 0.1M perchloric acid. Dopamine and the metabolites DOPAC and HVA were analysed by high performance liquid chromatography (HPLC). The HPLC system used was an Agilent 1100 Series system (Agilent Technologies, Inc., CA, USA) containing a temperature- controlled autosampler. Mobile phase consisted of 75mM NaH_2_PO_4_, 1.4mN octane sulfonate, 1mM EDTA and 12% acetonitrile (adjusted to pH 4.13 using phosphoric acid). Mobile phase was delivered at a flow rate of 1mL/min to a Kinetex 5μm C18 column, 150mm x 4.6mm (Phenomenex Inc., CA, USA). Dopamine, DOPAC and HVA were separated using a Coulochem III electrochemical detector (ESA Laboratories, Inc., MA, USA). The detector settings were as follows: conditioning cell (Model 5020, ESA Laboratories, Inc.) at +300 mV, analytical cell (Model 5014B, ESA Laboratories, Inc.) with the first and second electrodes maintained at −150 and +250 mV, respectively. Data were quantified by calculating peak-height ratios for each specific analyte relative to the internal standard, deoxyepinephrine. Data were stored and processed with Chemstation software (Rev B.01.03, Agilent Technologies, Inc.).

### Statistical analyses

Statistical analysis was performed using SPSS statistics program (IBM, version 20). Locomotor activity is presented as the total distance travelled over the 2h of measurement following MK-801 or saline injection. Locomotor activity during the induction phase was analysed with a two-way repeated measures ANOVA with Day (Days 1–7) as within-subjects factors and Pre-treatment (RU486 or vehicle) and Drug treatment (MK-801 or saline) as between-subjects factors. Locomotor activity was further analysed with a one-way repeated measures ANOVA for each the vehicle- and RU486- pre-treated groups with Day (Days 1–7) as within-subjects factors and Drug treatment (MK-801 or saline) as between-subjects factors. To determine whether animals had sensitised, factorial ANOVA was performed on locomotor activity at challenge with treatment (MK-801 or saline) as the between-subjects factor. The effect of RU486 and MK-801 on corticosterone and neurotransmitter levels were analysed by factorial ANOVA. Repeated measures ANOVA were performed on corticosterone levels between the days of induction with Day (Days 1, 4, 7) as the within-subjects factor and Group as the between-subjects factor. Statistical significance was set at *p*<0.05 and when a main effect or interaction was found, post-hoc testing was performed using a Dunnett’s test, to correct for multiple comparisons.

## Results

### Behavioural effects of RU486 on induction of MK-801 sensitisation

**Fig 2:**
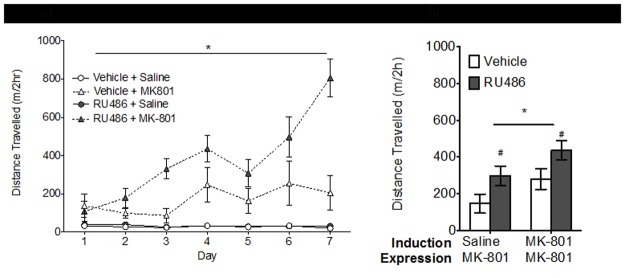
Effect of RU486 on MK-801 behavioural sensitisation. MK-801-induced locomotor sensitisation. (a) During the induction phase, rats received daily administration of RU486 (20mg/kg) *(grey symbols)* or DMSO vehicle *(white symbols)*. Thirty minutes later they were injected with MK-801 *(triangles)*(0.25mg/kg) or saline *(circles)* (n = 4-8/group). Locomotor activity is recorded as the distance travelled over 120min. Not surprisingly locomotion was increased by repeated MK-801 but not saline. RU486 had no effect on the saline exposed animals but produced an additive locomotor effect in the MK-801 treated animals that progressively increased across the induction phase (*p<0.05) (b) At the expression phase all animals received a single challenge dose of MK-801 (0.25mg/kg) and again distance travelled over 120min was measured. Rats previously treated with MK-801 displayed a sensitised locomotor response compared to saline treated controls (*p<0.05). Expression of MK-801-induced sensitisation was preserved in rats previously exposed to RU486. Overall, rats previously treated with RU486 *(grey bars)* during the induction phase displayed a heightened response to the MK-801 challenge (#*p*<0.05) compared to vehicle treated controls *(white bars)*. Data are presented as the mean ± SEM.

Fig 2A shows the locomotor response following MK-801 or saline injection across Days 1–7 of the induction phase. Although RU486 had no effect on locomotion itself, pre-treatment with RU486 elevated MK-801-induced locomotor activity. A repeated measures ANOVA revealed significant effects of Day (*F*_6,120_ = 6.79, *p*<0.001), Pre-treatment (RU486) (*F*_1,20_ = 9.53, *p*<0.01), Drug treatment (MK-801) (*F*_1,20_ = 49.56, *p*<0.01), and a Pre-treatment (RU486) x Drug treatment (MK-801) interaction (*F*_1,20_ = 8.72, *p*<0.01). Further analysis indicated that in the vehicle pre-treated rats, MK-801-induced locomotor activity was increased compared to saline-induced locomotor activity (*F*_1,6_ = 30.3, *p*<0.001). In the vehicle pre-treated rats there was no significant Day x Drug treatment interaction, indicating that MK-801-induced locomotor did not increase over the induction phase (Fig 2A). In the RU486 pre-treated rats, MK-801-induced locomotor activity was increased compared to saline-induced locomotor activity (*F*_1,14_ = 55.88, *p*<0.001). In the RU486 pre-treated rats, repeated measures ANOVA revealed a significant Day x Drug treatment interaction (*F*_1,14_ = 59.5, *p*<0.001). This indicates that RU486 pre-treatment selectively and progressively increased MK-801-induced locomotor activity over the induction phase.

### Behavioural effects of prior RU486 treatment on expression of MK-801 sensitisation

As expected, when rats were challenged with MK-801 after 5 days of drug withdrawal, the locomotor response was higher in the prior MK-801 treated rats compared to their saline controls (*F*_1,20_ = 5.12, *p*<0.05) (Fig 2B). No significant interaction between RU486 pre-treatment and MK-801 treatment was identified on the locomotor response to MK-801 challenge. This indicates that co-administration of RU486 with MK-801 during the induction phase, did not block the expression of MK-801 sensitisation as expected. Instead, as shown in Fig 2B, prior treatment with RU486 produced an enduring enhancing effect on the locomotor sensitivity to MK-801 challenge (*F*_1,20_ = 6.5, *p*<0.05), even after a withdrawal period of 5 days.

### Plasma corticosterone levels at induction

**Fig 3:**
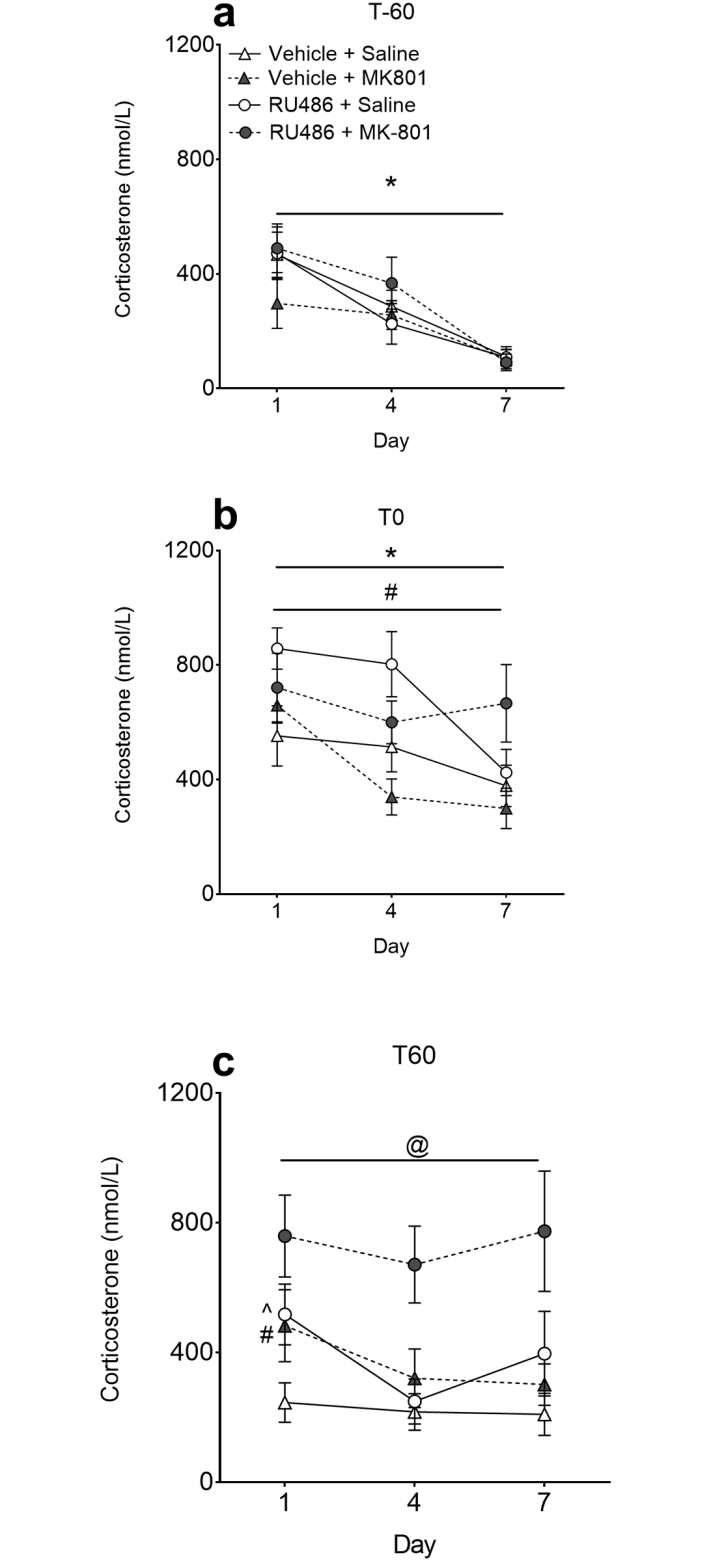
Plasma corticosterone at induction. Plasma corticosterone levels throughout induction were measured every third day. (a) Rats (n = 12/group) were bled prior to treatment (T-60) for baseline measures. Corticosterone levels at baseline decreased over the 7-day induction period. (b) After 30min habituation to the chamber half the animals were injected with RU486 and half with vehicle. Animals were then bled 30min later (T0). At this time point RU486 was shown to increase plasma corticosterone levels compared to vehicle groups across all days. A main effect of Day was also observed at this time point. (c) Immediately after being bled at T0 rats were injected with MK-801 (or saline) and a final blood sample was collected 60min later (T60). At the T60 time point on Day 1 there was a main effect of both RU486 and MK-801. A post-hoc Dunnett’s test on the repeated measures ANOVA indicate that the RU486+MK-801 group had significantly elevated plasma corticosterone levels across all days. *p<0.05 main effect of Day; # *p*<0.05 main effect RU486 pre-treatment; ^ *p*<0.05 main effect MK-801 drug treatment; @ *p*<0.01 RU486+MK-801 vs Vehicle+Saline. Data are presented as the mean ± SEM.

Blood was collected from a second cohort of rats for corticosterone analysis on Days 1, 4 and 7 of induction (protocol depicted in Fig 1B). Baseline measures of corticosterone (T-60) decreased with each subsequent day of sampling (*F*_1,36_ = 94, *p*<0.001) likely indicating a habituation to the procedure (Fig 3A). At T0 (30min after RU486 or vehicle injection) there was a significant effect of Day on corticosterone levels (*F*_1,30_ = 15.7, *p*<0.001). This decrease across days is further indication of habituation to procedure. As shown in Fig 3B, RU486 significantly *increased* corticosterone levels (*F*_1,30_ = 14.3, *p*<0.001). At T60 (60min after MK-801 or saline injection) on Day 1, corticosterone levels were significantly increased by MK-801 (*F*_1,33_ = 4.2, *p*<0.05) and RU486 (*F*_1,33_ = 7.1, *p*<0.05). This effect by each individual drug was no longer observed on Day 4 and Day 7. Repeated measures ANOVA on T60 corticosterone levels indicated no significant effect of Day but a significant effect of RU486 Pre-treatment (*F*_1,33_ = 10.6, *p*<0.01) and MK-801 Drug treatment (*F*_1,33_ = 7.1, *p*<0.05). A post-hoc Dunnett’s test revealed that only corticosterone levels in the RU486+MK-801 group were significantly higher than the Vehicle+Saline control group (*p*<0.01, Fig 3C). Thus, when given together, the two antagonists had an additive effect on corticosterone levels on the first day of induction. However, as there is no individual effect of each antagonist on Day 4 and 7 of the induction phase, the nature of the interaction has been altered.

### Plasma corticosterone levels at expression

**Fig 4:**
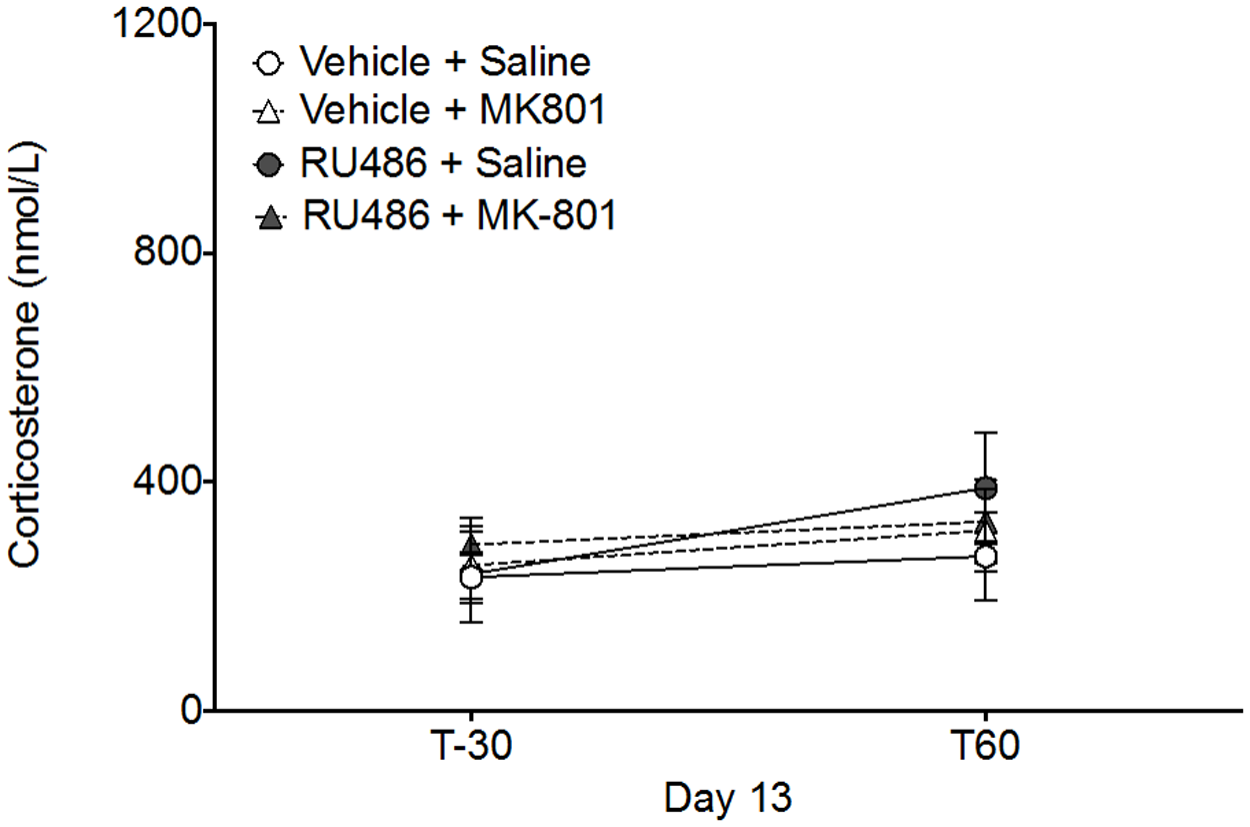
Plasma corticosterone at expression. Plasma corticosterone levels at the expression phase. All groups (n = 12) were habituated to the chamber for 30 min prior to receiving a challenge injection of MK-801 at T0. Corticosterone levels were unaltered by any prior treatment either at baseline (T-30) or 60min after MK-801 challenge (T60). Corticosterone levels did not significantly increase from baseline and were not affected by prior induction treatment of RU486 or MK-801. Data are presented as the mean ± SEM.

The corticosterone levels measured during the expression of sensitisation on Day 13 are depicted in Fig 4. At baseline (T-30) there was no significant effect of either RU486 Pre-treatment (*F*_1,43_ = 0.26, *p* = 0.609) or MK-801 Drug treatment (*F*_1,43_ = 0.17, *p* = 0.679) on corticosterone levels. Administration of an MK-801 challenge injection (T0) after 5 days washout had no effect on corticosterone levels when measured at T60. There was no significant effect of RU486 Pre-treatment (*F*_1,43_ = 0.69, *p* = 0.408) or MK-801 Drug treatment (*F*_1,43_ = 0.07, *p* = 0.933) on corticosterone levels measured at T60 on Day 13.

### Neurotransmitters and metabolites in the nucleus accumbens

**Fig 5:**
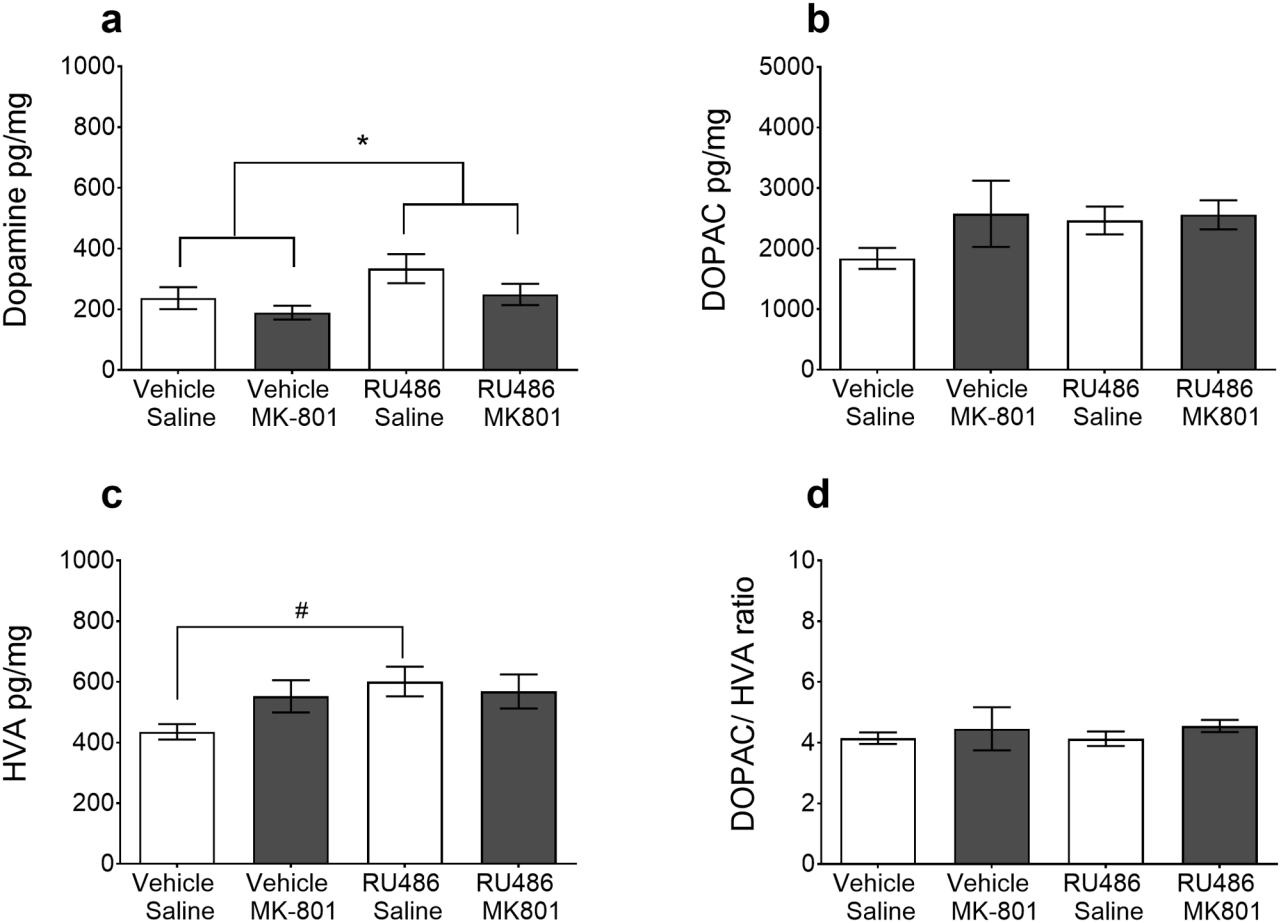
Dopamine and metabolite levels in the NAc. Nucleus accumbens tissue samples were collected one day after the final MK-801 challenge (n = 12/group). Dopamine (a) and its major metabolites DOPAC (b) and HVA (c) were significantly elevated in RU486 pre-treated rats *(grey bars)*. Since DOPAC and HVA were both increased there was no difference in the DOPAC/HVA ratio between groups (d). Data are presented as the mean ± SEM *p<0.05 RU486 pre-treatment effect (Two-way ANOVA) # *p*<0.05 compared with the Vehicle-Saline control rats in the post-hoc Dunnett’s test.

Dopamine and its metabolites were measured one day after MK-801 challenge in the NAc. Two-way ANOVA revealed that RU486 pre-treated animals had significantly increased dopamine levels (*F*_1,44_ = 4.6, *p*<0.05) in the NAc compared to vehicle controls (Fig 5A). This increase in dopamine was not affected by prior MK-801 treatment. The post-hoc Dunnett’s test revealed that compared to the Vehicle+Saline control group, HVA (Fig 5C) tissue levels were significantly elevated in the RU486+Saline group (*p*<0.05).

## Discussion

This study investigated the role of GR signalling in a model of MK-801 sensitisation in male Sprague Dawley rats. This was achieved by co-administration of the GR antagonist RU486 with MK-801 during the induction phase. In this study we found that RU486-treated rats did not differ from vehicle controls in the initial response to MK-801 injection. However, there was a progressive increase in the locomotor response across the induction phase and, contrary to our initial hypothesis, prior treatment with RU486 did not block the development of MK-801 sensitisation. In addition to the augmenting effect of RU486 on MK-801-induced behaviour during induction, RU486 was found to have an additive effect on MK-801-induced plasma corticosterone levels.

Based on previous studies demonstrating a role for glucocorticoids in behavioural sensitisation to other psychostimulants, including cocaine [30], amphetamine [16], and ethanol [18], it was hypothesised that the co-administration of the GR antagonist would block the development of MK-801 sensitisation. The present results did not support this hypothesis. These results also contradict those of the one study to date that investigated GR function in MK-801 sensitisation [26]. The authors reported that when RU486 was given in conjunction with a single MK-801 administration, the sensitised response to the MK-801 challenge 48h later was inhibited [26]. The most critical difference between the present study and that of Wedzony and Czyrak [26], is that our study implemented a *repeated* MK-801 exposure paradigm during induction rather than a single MK-801 exposure. The effects of RU486 may differ significantly between acute and chronic administration of the NMDAR antagonist. The differences between the effects of RU486 on MK-801 sensitisation between the two studies could also be attributed to the use of different rodent strains. The acute MK-801 sensitisation study by Wedzony and Czyrak (1994) utilised male outbred albino Wistar rats, whereas our study utilised male outbred albino Sprague Dawley rats. Although these are both outbred albino rat strains, the role of corticosteroids in drug-induced behavioural sensitisation has previously been shown to be strain-dependent [31]. Previous reports have demonstrated that Wistar and Sprague Dawley rats display differential behavioural, neurochemical and physiological responses to stress [32]. It is therefore plausible that differential activation of the GRs may be required for development of sensitisation to MK-801 in Wistar compared with Sprague-Dawley rats.

Acute administration of RU486 had potentiating effects on acute corticosterone production. This is consistent with previous reports of the effects of RU486 on corticosterone levels [18, 33]. This is thought to occur via disinhibition of the negative feedback of corticosterone from higher components of the HPA axis [34]. Corticosterone synthesis is self-regulated through suppression of corticotropin-releasing hormone (CRH). Blocking GRs with RU486 would attenuate this negative feedback, allowing CRH to stimulate the release of adrenocorticotropic hormone (ACTH), which results in increased corticosterone synthesis [34]. Consistent with previous studies [18, 35], the present study demonstrated that repeated administration of RU486 resulted in a tolerance-like response to its corticosterone-inducing effects.

Congruent with past reports, MK-801 was also shown to acutely increase corticosterone levels [13, 26, 36]. With repeated exposure however this lead to a tolerance-like response, which has also previously been demonstrated [36]. The precise mechanism by which MK-801 stimulates the secretion of corticosterone is still unclear. MK-801 has been shown to increase the biosynthesis of CRH from the parvocellular neurons of the paraventricular nucleus (PVN) in the hypothalamus [37] and to stimulate the release of ACTH [36]. The stimulatory effects of NMDAR antagonism on the HPA axis are therefore centrally mediated. This is further supported by early findings demonstrating that the NMDAR antagonist PCP has no direct stimulatory effects at the level of the pituitary or adrenal glands [38].

Consistent with its effect of increasing MK-801-induced locomotor behaviour, RU486 also potentiated the corticosterone-inducing effects of MK-801. When either antagonist was given alone (i.e. RU486+Saline or Vehicle+MK-801) corticosterone was only elevated on Day 1 of induction. In contrast, the combined administration of RU486 and MK-801 lead to sustained increased levels of plasma corticosterone throughout induction. RU486 has previously been reported to produce a similar pattern of potentiated corticosterone- production when combined with other drugs such as ethanol [18]. Despite this enhancing effect on ethanol-induced corticosterone, Roberts et al. [18] reported that, in mice, the co-administration of RU486 with ethanol for 10 days blocked the development of a sensitised locomotor response to the ethanol challenge one day later. Unfortunately this study did not monitor locomotor activity during the induction phase. Thus it is unknown whether the augmenting effect RU486 had on ethanol-induced plasma corticosterone during induction was also reflected in behavioural locomotor activity. It is well known that ethanol, in addition to stimulating GABA_A_ receptor function [39], can also inhibit the function of NMDARs [40, 41]. Together with the results of Roberts et al., (1995), our results suggest that the potentiating effect of RU486 on MK-801-induced corticosterone release may be due to an effect of RU486 on the NMDARs and/or glutamatergic transmission.

Though locomotor sensitisation to MK-801 was present at the expression phase, there was no sensitised corticosterone response. This finding is consistent with previous reports demonstrating that repeated ethanol [18] or cocaine [42, 43] administration sensitises the behavioural, but not the neuroendocrine response. In our study prior RU486 treatment had long-term effects on locomotor behaviour (observed after MK-801 challenge), but not corticosterone levels. The effect of RU486 on augmenting MK-801-induced corticosterone levels is evidently short-term, since it is dependent on the presence of RU486. An increase in corticosterone levels however cannot explain the longer-term effects of RU486 on the sensitivity to the locomotor activating effects of MK-801.

Increased dopamine release in the NAc during challenge has been demonstrated in MK-801 sensitised animals by microdialysis [6]. In the vehicle pre-treated animals that were sensitised to MK-801, tissue levels of dopamine and its metabolites in the NAc were not increased when measured 24h after exposure to MK-801. However, dopamine tissue levels all increased in animals that had previously been treated with RU486 during the induction phase, even after 6 days withdrawal. Tissue levels of dopamine typically reflect presynaptic storage. Thus, it is plausible that, when challenged with MK-801, RU486 pre-treated animals had a greater available pool of dopamine within the NAc that could be released, thereby increasing the hyperlocomotor response to MK-801. The dopamine metabolite HVA was significantly increased only in the RU486+Saline treated group indicating dopamine turnover was increased selectively in this group. It is therefore possible that a dopaminergic mechanism also underlies the enhanced locomotor response to MK-801 challenge in the RU486 treated rats.

To the best of our knowledge this is the first study to show that repeated RU486 administration induces a persistent increase in dopamine levels in the NAc. Substantial evidence indicates that glucocorticoid hormones regulate the mesolimbic dopamine system [44]. The mechanism by which chronic GR antagonism results in increased dopamine is currently unknown. One potential mechanism for the increase in dopamine and metabolites observed in this study is through increased expression of tyrosine hydroxylase (TH), the rate-limiting enzyme in catecholamine synthesis [45]. Through a glucocorticoid-responsive element on the promoter region of the TH gene, corticosterone has been shown to directly increase TH transcription [46, 47]. Thus, in the present study, RU486 induced corticosterone release occurring during the induction phase could exert long-term genomic effects on TH mRNA. Because an increase in TH could produce the long-term increase in dopamine and its metabolites in the NAc further investigation into this mechanism is warranted.

In summary, our data indicates that the GRs are a potential moderator in the development of MK-801-induced locomotor sensitisation. The increased dopamine tissue levels induced by RU486 are suggestive of a potential mechanism involved in the interaction between RU486 and MK-801. Future studies could address this through pharmacological manipulation of the dopamine receptors or 6-OHDA lesions of the dopaminergic terminals. MK-801 behavioural sensitisation is purportedly independent of dopamine release and occurs in 6-OHDA lesion animals [48]. Therefore co-administration of RU486 in 6-OHDA lesion animals during the induction of MK-801 sensitisation, could determine if the effect of RU486 is dependent on dopamine release. Furthermore, it was shown that the corticosterone inducing effects of the NMDAR antagonist MK-801 is potentiated by GR inhibition, similar to the effects observed with ethanol. Although ethanol also has some NMDAR antagonist properties [49], the manner in which stress signalling contributes to ethanol sensitisation is analogous to the dopaminergic psychostimulants rather than to MK-801. Overall these findings indicate there is a clear distinction between mechanisms related to behavioural sensitisation to MK-801, compared to behavioural sensitisation to other drugs of abuse. It is important to note that this study was conducted only in male rats and further study would need to be conducted to determine if the same results would be obtained in female rats. The GR antagonist RU486 used in this study, has also been used in clinical trials for treatment of a range of neuropsychiatric disorders [50, 51] including schizophrenia [52, 53] and addiction [54]. The results of this study suggest that RU486 would not likely be useful in the treatment of individuals who abuse NMDAR antagonists such as PCP and ketamine. In addition, this raises the critical question of how NMDAR dysfunction in schizophrenia would be affected, possibly even potentiated, by clinical treatment with RU486.

## Supporting information

S1 Table Raw locomotor behavior data. (XLSX)

S2 Table Raw plasma corticosterone levels. (XLSX)

S3 Table Raw HPLC dopamine, DOPAC and HVA data. (XLSX)
